# Multi-Sensory Tool Holder for Process Force Monitoring and Chatter Detection in Milling

**DOI:** 10.3390/s24175542

**Published:** 2024-08-27

**Authors:** Alexander Schuster, Andreas Otto, Hendrik Rentzsch, Steffen Ihlenfeldt

**Affiliations:** Fraunhofer Institute for Machine Tools and Forming Technology (IWU), 09126 Chemnitz, Germany; andreas.otto@iwu.fraunhofer.de (A.O.); hendrik.rentzsch@iwu.fraunhofer.de (H.R.); steffen.ihlenfeldt@iwu.fraunhofer.de (S.I.)

**Keywords:** smart tool holder, sensor integrated, process monitoring, chatter, cutting force, vibration

## Abstract

Sensor-based monitoring of process and tool condition in milling is a key technology for improving productivity and workpiece quality, as well as enabling automation of machine tools. However, industrial implementation of such monitoring systems remains a difficult task, since they require high sensitivity and minimal impact on CNC machines and cutting conditions. This paper presents a novel multi-sensory tool holder for measurement of process forces and vibrations in direct proximity to the cutting tool. In particular, the sensor system has an integrated temperature sensor, a triaxial accelerometer and strain gauges for measurement of axial force and bending moment. It is equipped with a self-sufficient electric generator and wireless data transmission, allowing for a tool holder design without interfering contours. Milling and drilling experiments with varying cutting parameters are conducted. The measurement data are analyzed, pre-processed and verified with reference signals. Furthermore, the suitability of all integrated sensors for detection of dynamic instabilities (chatter) is investigated, showing that bending moment and tangential acceleration signals are the most sensitive regarding this monitoring task.

## 1. Introduction

The rapid development of digital technologies and the “Industrial Internet of Things” (IIoT) are the driving forces behind the ongoing industrial revolution known as “Industry 4.0”. As part of this revolution, advanced manufacturing techniques are combined with IIoT systems with the goal of achieving automated, unmanned production. Important components in this context are intelligent monitoring systems that provide information about machine, process and tool conditions. Monitoring is particularly crucial in highly value-adding machining processes, where small errors have a direct impact on the overall productivity and quality of the final product [1,2]. One common but considerably challenging error is the changing tool condition, especially the gradually progressing wear of cutting edges. Depending on workpiece, tool and machining conditions, different wear types occur, e.g., flank wear, crater wear, notch wear, plastic deformation, edge chipping, thermal cracks or built-up edges [3,4]. Generally, tool wear results in reduced accuracy and surface quality of the manufactured workpieces, ultimately leading to production rejects. Critical wear levels can also lead to tool breakage and, hence, costly machine downtime [5,6]. Another challenging error in industrial manufacturing is the dynamical instability of milling processes, also referred to as chatter [7,8]. This effect occurs during the regenerative excitation of critical natural frequencies of a mechanical system due to the periodic engagement of the tool edges with the workpiece material, resulting in significantly increased relative vibrations between tool edges and the workpiece. Consequences of chatter include poor surface quality and inaccuracy of workpieces, excessive noise and accelerated wear of tools and machine tool components, such as spindle bearings [7].

In order to reliably prevent these events, sensitive monitoring systems are essential. In particular, continuously measuring in-process sensor systems are of great interest, as they allow for real-time detection and avoidance of errors, therefore not requiring intermittent production stops to directly measure, for example, tool wear or scrapped parts [2,6]. These sensor systems are typically combined with corresponding signal processing algorithms and analysis models to establish the relationship between measured signals and monitoring scopes. Teti et al. [1], Shokrani et al. [2], Bleicher et al. [9], Kuntoğlu et al. [10] and Mohamed et al. [11] each recently presented a comprehensive review on monitoring systems based on this data-driven approach. These papers concluded that cutting force and torque, motor current and power, vibrations, temperature and acoustic emission (AE) are the most significant sensor signals for process and tool condition monitoring. The associated sensors have been integrated in spindle and axis drives, machine frame components, the spindle periphery, workpiece tables, workpiece fixtures or directly on the tool holder or tool. Due to the variety of potential machine tool configurations, tools, machining strategies and sensor application locations, the measurement ranges of the previously mentioned parameters differ significantly. A few examples are presented in [10].

It has been found that the ability to detect small changes in a process increases when sensors are mounted closer to the process zone [5,10]. Therefore, sensor integration on the tool holder is a suitable approach to achieve maximum sensitivity in process monitoring. Although the tools themselves are even closer to the process, they have a limited lifespan due to wear, making sensor integration uneconomical in such cases. A comprehensive overview of sensor-integrated tool holders was reported by Shokrani et al. [2] and Mohamed et al. [11]. Due to the rotation of the tool holder, power supply is realized either by batteries, inductive coupling between a stationary primary coil and a secondary coil located on the rotor or via a slip-ring contact between the tool holder and stator component. These solutions either result in a limited operating time, preventing their application in series production, or they come with significant interfering contours on the tool holder and spindle, as well as cables in the machine’s working area. Furthermore, the integration of sensors and electronics in almost all designated systems leads to longer tool-holder lengths; geometries protruding beyond the tool-holder contour; and, in some cases, significantly reduced stiffness. These aspects increase the integration effort on the one hand and imply collision risks and restrictions for regular machine operation on the other hand, which is considered unfavorable for use in an industrial environment [11]. These points are seen as deficits in the state of research. The smart tool holder developed by the authors and presented here aims to overcome these disadvantages through wireless data transmission and a novel self-sufficient power supply technology based on electromagnetic induction. Moreover, the system is equipped with multiple sensors in order to detect a broad range of potential machining errors. The fundamental functionality of these sensors, as well as their suitability for sensitive process monitoring with a focus on chatter detection, is examined in this work.

This paper is organized as follows. The methodology, i.e., sensor integration on a tool holder, as well as energy supply and data transmission technology, is described in Section 2. The experimental setup, cutting parameters and observations are presented in Section 3. The results of the cutting tests with focus on verification of sensor signals and detection of chatter are presented in Section 4, followed by a discussion and conclusions in Section 5.

## 2. Methodology

### 2.1. Sensor Integration on Tool Holder

As described in the previous section, in numerous recent papers, different sensor types applied to various machine tool components have been tested and evaluated regarding their applicability for process and tool condition monitoring. Extensive information about tool wear, breakage, chipping and chattering during milling processes, as well as surface roughness and dimensional accuracy of the machined workpieces, can be provided by force and vibration signals [1,2]. Therefore, the smart tool holder presented in this paper follows a multi-sensor approach by incorporating both strain gauges for cutting force measurement and a piezoelectric accelerometer for monitoring of vibrations.

**Figure 1:**
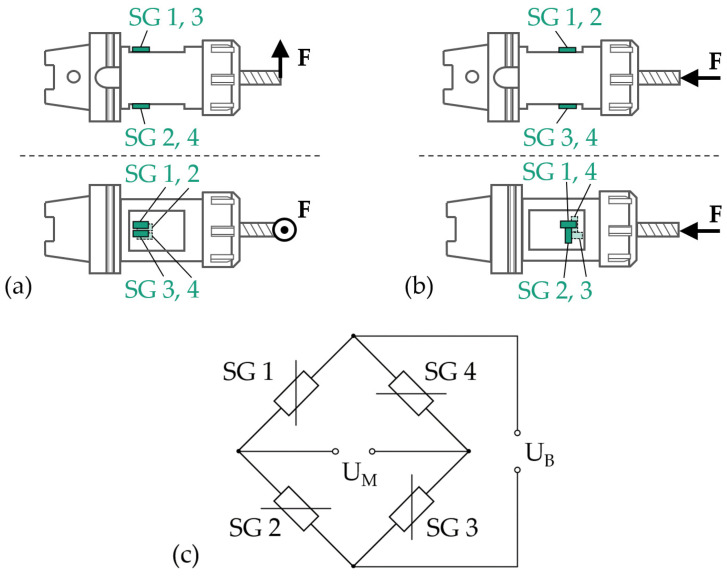
Application of strain gauges (SGs) to a tool holder for measurement of (**a**) force F in radial direction (bending moment) and (**b**) force F in axial direction; (**c**) connection of strain gauges to Wheatstone full-bridge circuits.

Linear metal foil strain gauges (1-LM11-3/1K0GE) from HBM (Darmstadt, Germany) are selected in this work. The grid material is a 5 μm thick nickel–chromium alloy (“Modco”) that is carried by 35 ± 10 μm thick glass fiber-reinforced phenolic composites. This strain-gauge type comes with integrated strain-relieved solder pads, a nominal resistance ($R_{0}$) of 1 kΩ at 23 °C and a gauge factor (k) of approximately 2.2. The fatigue life of the selected strain gauge is >$10^{7}$ load cycles at a strain amplitude of 2000 μm/m with maximum zero-point drift of ±100 μm/m. The maximum elongation of the sensor is 10,000 μm/m in the positive direction and 15,000 μm/m in the negative direction. The range of operating temperature is −200–250 °C, and the temperature response tolerance of the sensor is ±0.3 ppm/K. Several of these linear strain gauges are applied to the tool holder’s surface to measure the strains that result from cutting forces acting on the tool. The relative change in resistance ($r_{i}$) of a single strain gauge (i) is expressed as [12]

(1) $$r_{i}=\frac{∆R_{i}}{R_{0}}=k\varepsilon_{i},$$

where $∆R_{i}$ is the absolute change in resistance and $\varepsilon_{i}$ is the strain at the application surface and, thus, the strain of the sensor. Since tool holders typically require high stiffness to minimize tool deflection and ensure sufficient dimensional accuracy of machined workpieces, strain occurring on the tool-holder surface is limited to small values. The corresponding changes in the sensors’ resistance and the potential electrical output signals are modest as well. Additionally, the change in resistance depends on several external factors, e.g., temperature. Therefore, four strain gauges are commonly connected to Wheatstone full-bridge circuits to compensate for many of those errors and to amplify the output signals. The simplified bridge equation is expressed as [12]

(2) $$\frac{U_{M}}{U_{B}}=\frac{1}{4}\left(r_{1}-r_{2}+r_{3}-r_{4}\right)$$

where $U_{B}$ is the bridge supply voltage and $U_{M}$ is the measurement signal (bridge output voltage). The application of the four strain gauges in specific relative orientations on the tool-holder surface leads to either summation or elimination of the relative changes in resistance of the individual strain gauges within the bridge circuits. This enables measurement of different directional components of the cutting force. The proposed sensory tool holder is equipped with two full bridges. One is sensitive to strains from radial forces, and the other one is sensitive to axial forces. Since the length of cutting tools typically varies, the radial force component is more conveniently monitored as a bending moment that is independent of the cutting-tool length. In Figure 1, the realized full-bridge circuits and sensor orientations are presented. The sensor application, as well as the corresponding pockets, is rotationally symmetric to prevent static or dynamic imbalances of the tool holder. For bending moment measurement, equally strained sensors are interconnected in opposite bridge circuit arms, and inversely strained sensors are interconnected in adjacent bridge circuit arms. All strains are equal regarding amount when a bending moment is acting on the tool ($\varepsilon_{1}=-\varepsilon_{2}=\varepsilon_{3}=-\varepsilon_{4}$ with $\varepsilon_{b}=\varepsilon_{1}$). In the case of the axial force-sensing full-bridge circuit, strain gauges 1 and 3 are interconnected in opposite bridge-circuit arms and strained in equal directions and with equal amounts when axial forces are acting on the tool ($\varepsilon_{1}=\varepsilon_{3}$ with $\varepsilon_{ax}=\varepsilon_{1}$). Strain gauges 2 and 4 are interconnected in adjacent bridge arms to strain gauges 1 and 3. These sensors detect the lateral strain of an equal amount and direction ($\varepsilon_{2}=\varepsilon_{4}$, with $\nu \varepsilon_{ax}=\varepsilon_{2}$ and ν being Poisson’s ratio of the deformed body). Poisson’s ratio (ν) depends on the actual structural design of the tool holder and, thus, is determined via static–mechanical analysis with finite element analysis (FEA) using Ansys Workbench 2021 R2 software. In detail, an external axial force of 1 N is applied to the tool tip, and the resulting longitudinal and lateral strains at the strain-gauge application positions are calculated. The ratio between the longitudinal and lateral strains equals the Poisson ratio. By inserting Equation (1) and the described strain relations into Equation (2), the following equations are established for conversion between the electrical output signal ($U_{M}$) and principal strains caused by the bending moment ($\varepsilon_{b}$) and axial force ($\varepsilon_{ax}$).

(3) $$U_{M}=k\varepsilon_{b}U_{B}$$

(4) $$U_{M}=\frac{1}{2}k\left(1+\nu \right)\;\varepsilon_{ax}U_{B}$$

An important feature of these full-bridge circuits is their behavior when strain occurs that is caused by forces with directions different from those to be measured. The bending moment sensing bridge circuit is insensitive to strains from axial forces or torque. The axial force sensing bridge circuit is insensitive to strains from radial forces or torque. Both full-bridge circuits are insensitive to changes in sensor resistance due to varying temperature if they are equal for all four strain gauges of each bridge. In all these cases, the relative change in resistance of the four sensors is compensated for within the bridge circuits, i.e., $U_{M}=0$. The dependence of the sensors’ sensitivity on temperature is mostly compensated through additional temperature-dependent resistances within the bridge circuits. The dependence of the nominal bridge output voltages on temperature due to the varying lengths of bridge supply wires, small dimensional errors of the tool holder and sensor application or changes of the insulation resistance are also mostly compensated for through additional temperature-dependent resistances. The remaining thermal signal deviations after compensation are marginal in relation to the expected signal values and, hence, not further determined.

Equations (3) and (4) define the linear conversion between strain and bridge output signal. Since Hooke’s law describes a linear relation between force and strain in the case of linear-elastic deformation, a linear relation between output signals and forces also applies. The respective load-to-strain relations for bending moment and axial force heavily depend on the structural design of the tool holder. Therefore, proportionality factors $\Pi_{b}$ and $\Pi_{ax}$ are introduced to express these relations and enable conversion between electrical output signals and force quantity.

(5) $$\varepsilon_{b}=\Pi_{b}M_{b}$$

(6) $$\varepsilon_{ax}=\Pi_{ax}F_{ax}$$

These factors are initially determined via FEA, equally to Poisson’s ratio, then practically calibrated by applying static radial and axial forces on the tool tip of the sensory tool holder while measuring the exact forces with a spring scale. The final equations for conversion between forces and bridge output voltage are derived by inserting Equations (5) and (6) into (3) and (4), respectively.

(7) $$U_{M}\left(M_{b}\right)=k\Pi_{b}U_{B}M_{b}$$

(8) $$U_{M}\left(F_{ax}\right)=\frac{1}{2}k\left(1+\nu \right)\;\Pi_{ax}U_{B}F_{ax}$$

The corresponding parameters are presented in Table 1.

**Figure 2:**
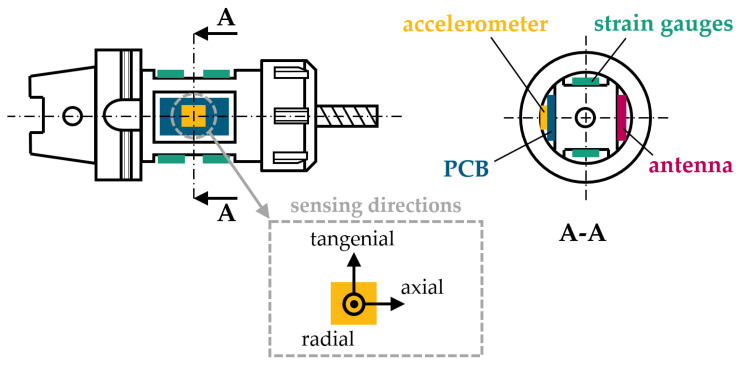
Integration of a piezoelectric accelerometer on the PCB of a smart tool holder in side view and cross-sectional view A-A, and the orientation of its sensing directions.

In addition to strain gauges for cutting force measurement, an acceleration sensor is integrated in the proposed smart tool holder (see Figure 2). Due to the variety and complexity of cutting processes, vibrations typically occur across several degrees of freedom. Therefore, an 830M1-0100 piezoelectric accelerometer from TE Connectivity (Berwyn, IL, USA) with three orthogonal sensing directions is selected. With a sensitivity of 12.5 mV/g at 20 °C and a measurement range of ±100 g for each direction, the signal output range equals ±1.25 V. The sensor is capable of measuring vibrations in the range of 2–15,000 Hz. It has a sensing non-linearity of ±2% and an operating temperature range of −40–125 °C. The shift in sensor sensitivity caused by thermal influences is approximately linear with 0.1 %/°C. Above 100 °C, the shift in thermal sensitivity is non-linear, which leads to a sensitivity deviation of approximately 7.5% at 125 °C. This sensor is directly mounted on the printed circuit board (PCB) in a separate pocket next to the strain gauges. The sensing directions are radially, axially and tangentially oriented. MEMS (“micro-electro-mechanical system”) sensors are another common accelerometer type besides piezoelectric accelerometers. They contain mechanical micro-structures that are combined with electronic components. Therefore, the motion of the mechanical structures is converted into an electrical output signal. Special characteristics of this accelerometer type include its ability to measure static accelerations. However, in our application, the tool holder is designed for spindle speeds up to 20,000 rpm corresponding to static centrifugal accelerations at the sensor location of up to 9000 g. This would require a MEMS sensor with a total measurement range of ±10,000 g, which heavily decreases the sensor’s sensitivity and, therefore, its ability to detect dynamic accelerations originating from the machining process around the comparably high static acceleration from tool rotation. To compensate for this disadvantage, MEMS sensors can also be integrated directly on the rotational axis, where radial accelerations from centrifugal forces become zero. Examples of such a sensory tool holder design were presented by Bleicher et al. [13] and Xie et al. [14]. As shown in these papers, this approach for MEMS accelerometer integration leads to an inevitable reduction in the tool holder’s cross section and, thus, stiffness reduction, as well as additional manufacturing effort for relocation of the internal hole to supply cooling lubricant. Due to these downsides, the sensory tool holder presented in this work follows a novel approach by utilizing a piezoelectric accelerometer. Sensors based on the direct piezoelectric effect generate an electrical charge when being stressed with mechanical load. This electrical charge on the piezoelectric element’s surface is neutralized within a short time. Therefore, statically applied forces and accelerations cannot be sufficiently detected [15]. In contrast, dynamically changing mechanical loads are measured with high sensitivity. These unique characteristics enable eccentrical integration on the rotating system.

In addition to the strain gauges and piezoelectric accelerometer, a resistive Pt1000 temperature sensor with a nominal resistance of 1000 Ω at 0 °C, a temperature coefficient of 3850 ppm/K, accuracy class AA according to [16] and a measurement range of −200–600 °C is applied onto the tool holder’s surface next to the strain gauges. The temperature signals are used to ensure compliance with hardware temperature limits and to enable compensation of residual thermal dependencies of the acceleration and strain sensors. This sensor signal is not intended for monitoring of process and tool conditions. All previously described sensor signals are filtered, amplified and digitalized by the tool holder-integrated PCB. Properties of the sensors and signal processing equipment are presented in Table 2.

The proposed high sampling rate of 10 kHz allows for detection of signals of up to 5 kHz according to the Nyquist-Shannon sampling theorem. This capability is enabled by operating the integrated microcontroller at maximum utilization.

### 2.2. Energy Supply and Data Transmission

**Figure 3:**
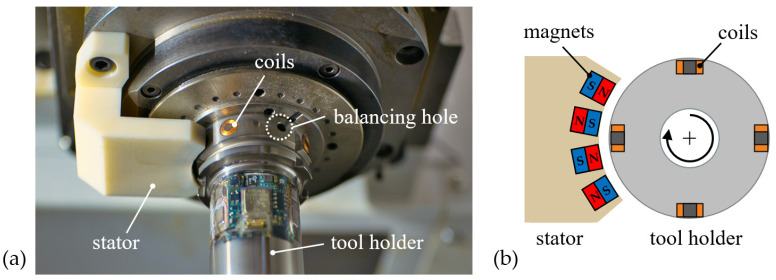
(**a**) Components of the electric generator for power supply of a sensory tool holder; (**b**) cross section of the stator and rotor sides of generator components.

Since wired energy supply and data transmission are not feasible for rotating components like cutting tools, the sensor-integrated tool holders mentioned in Section 1 are equipped with either batteries and wireless data communication based on radio transmission [14,17,18,19,20,21], an inductive energy and data transmission technology [22,23,24], or slip-ring technology for energy and data transmission [25]. Batteries lead to limited operating times and, thus, impede flexible usage in serial production. Inductive and slip-ring energy and data transmission introduces relevant overhanging components on both the rotor and stator sides, interfering with the working space of the machine tool. Moreover, the stator side requires an external energy supply via cables that must be mounted inside the critical working space. All solutions come with disadvantageous properties that are challenging regarding a permanent implementation in machine tool systems. In order to overcome these disadvantages, the energy supply of the smart tool holder proposed by the authors utilizes a novel electric generator. The principle is transformation of kinetic energy of the tool rotation into electrical energy through electromagnetic induction, thereby creating permanent independence from external energy supply. As depicted in Figure 3, four cylindrical coils with ferrite cores are integrated in pockets on the standardized HSK-A63 spindle interface of the tool holder (rotor) and connected to the PCB through internal cable ducts. No overhanging contours are caused by the coil integration. Alternately aligned magnets are integrated in a stator component that is mounted on the non-rotating spindle part. These magnets generate a permanent static magnetic field. During tool rotation, the coils move past the magnetic field originating from the stator. Thereby, electrical voltage is induced in the coils, which is processed to supply the smart tool holder’s hardware with energy. The compact designs of the stator and rotor still allow for automatic tool change and flexible usage of the system in serial production.

Wireless data communication is realized through radio transmission using the Bluetooth Low Energy (BLE) protocol. The corresponding transmitter antenna is integrated in a separate pocket that has identical dimensions as the pocket for the PCB and acceleration sensor but is diametrically opposed to it, as shown in Figure 2. All electronic components are covered with a compound of bisphenol-based epoxy resin and isophorondiamine hardener that provides sufficient chemical resistance to cooling lubricants used in machining. This compound is cured inside a temporarily attached silicon mold for over 24 h at room temperature. In this way, the outer design does not differ from standard tool holders and, thus, does not imply any interfering contours on the machine and process. The weight difference between the pocket with the antenna and the pocket with the PCB and accelerometer introduces dynamic and static imbalances. These imbalances are compensated for by balancing holes next to the coils (see Figure 3) and between the electronic section and collet chuck. Thereby, a balancing grade of G2.5 at a spindle speed of 25,000 rpm is achieved.

The digitalized sensor signals of the tool-holder system are sent in packages containing 32 consecutive values per signal. Due to the nature of the BLE connection, data packages are occasionally lost. The package loss rate heavily depends on the surrounding conditions of the receiver antenna, i.e., machine tool setup and mounting position. Additionally, as described in Section 2.1, the integrated microcontroller is operated at maximum utilization to achieve the presented high sampling rates. In our first prototype, this leads to the unintended effect of BLE packages being sporadically not sent, which will be fixed in future versions of the smart tool holder. Therefore, both the wireless connection and microcontroller utilization cause an occasional loss of signal sequences. In this way, on average, roughly 20 signal sequences are lost per second, and each lost signal sequence usually contains one to three data packages. This specific characteristic needs to be addressed when applying signal processing and analysis methods.

**Figure 4:**
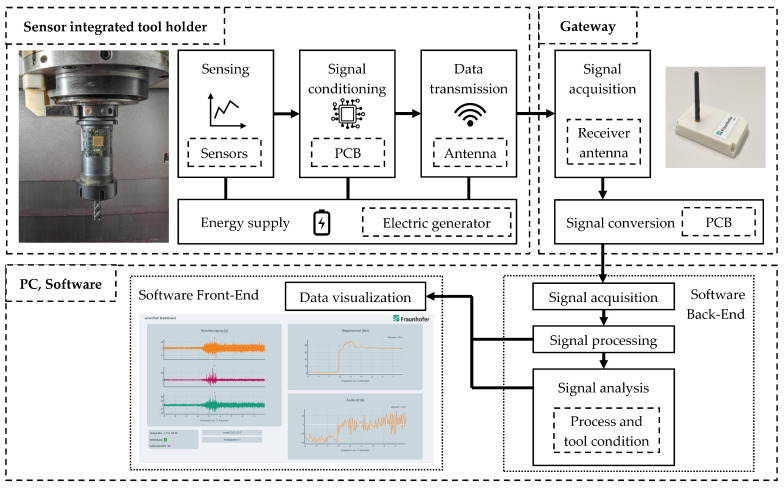
Scheme of the monitoring system based on the sensor-integrated tool holder; the arrows illustrate the data flow from the sensors to the software front-end.

The sensor data are received and converted by a gateway system. Its antenna is positioned at an arbitrary location inside the working space of the machine tool or right next to it. The gateway communicates with a PC unit that acquires, processes, analyzes and visualizes the incoming sensor signal packages. The structure of the monitoring system based on the sensor-integrated tool holder is presented in Figure 4.

## 3. Experimental Setup and Observations

### 3.1. Verification of Strain Gauge-Based Force Measurement

**Figure 5:**
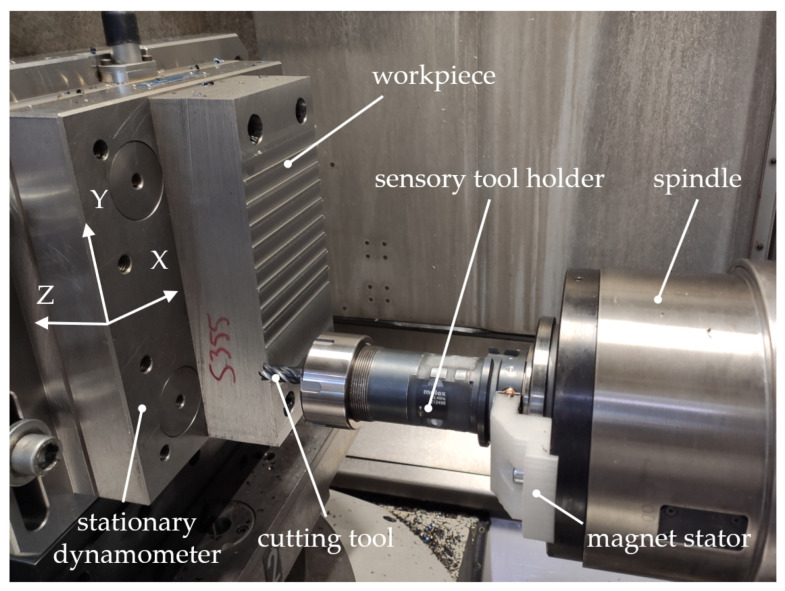
Experimental setup of tests for verification of force measurement.

Since the presented smart tool holder embodies a newly developed force transducer based on strain gauges applied to a customized tool-holder structure, verification of the realized bending moment and axial force measurement is necessary. As described in Section 2.1, the sensors (strain gauges in full-bridge circuits) are already statically calibrated with a spring scale. However, process forces during machining typically do not equal static loads. Thus, the force measurement of the tool-holder system is further investigated in cutting tests. These tests are conducted on a 5-axis HEC 500 D XXL milling machine from Starrag Heckert (Chemnitz, Germany). Several straight slots are cut in a workpiece made of S355JR steel with a cutting speed of 95 m/min, a depth of 1 mm and a width of 12 mm. A solid carbide end mill with a TiAlN coating, 12 mm diameter and four cutting edges is used without cooling lubricant. Since milling with this set of cutting parameters typically comes with only small axial forces, drilling tests are additionally conducted using the same workpiece material. A solid carbide drill with a TiAlN coating, 10 mm diameter and two cutting edges is used without cooling lubricant to drill holes of 20 mm depth with a cutting speed of 80 m/min. During the milling and drilling tests, only the feed rates are varied in order to achieve different process forces without significantly changing the cutting kinematics. The workpiece is mounted on a stationary 9255B dynamometer from Kistler (Winterthur, Switzerland) to measure process forces up to 5 kN in three orthogonal directions (X, Y and Z). These force signals are digitalized with a sampling rate of 12.8 kHz and serve as references for the force signals measured by the sensor-integrated tool holder. The experimental setup is shown in Figure 5.

In contrast to the strain gauge-based force measurement, the tool holder-integrated accelerometer does not require such in-process verification, as it is a self-contained sensor unit that is already calibrated by the manufacturer.

### 3.2. Experimental Setup for Process Monitoring Tests and Chatter Detection

**Figure 6:**
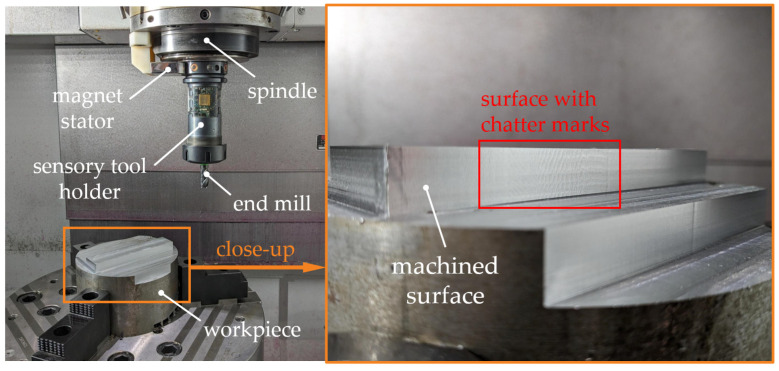
Experimental setup of milling tests for process monitoring with close-up view of the machined surface after a test with chatter.

For detailed investigation of the fundamental behavior of all tool holder-integrated sensors and their suitability for chatter detection, further cutting tests are carried out on a VMC 300 MT turn-milling center from EMAG (Salach, Germany). Down milling with varying process parameters is conducted using a workpiece made of S355JR steel without cooling lubricant. A new solid carbide end mill with a TiAlN coating, 12 mm diameter and 4 cutting edges is used. Different cutting depths are tested because chatter typically occurs above a certain critical depth of cut. Cutting speed is varied in order to investigate two different dynamical excitation frequencies of the mechanical structure and, thus, different dynamical behavior. The experimental setup and an exemplary machined surface with chatter marks are shown in Figure 6.

The tested combinations of cutting parameters, including a note regarding chatter occurrence, are presented in Table 3. Chatter is identified by measuring noise with a microphone and visually checking the machined surface for corresponding chatter marks. During execution of the tests, two aspects are observed.

1. Chatter in test 8 is considerably less intense than in test 6. This is identified by the magnitude of noise.
2. Chatter in test 4 only occurs in the second half of the cut, while the first half is dynamically stable. This indicates that the mechanical structure is less prone to chattering when milling with the higher selected cutting speed.

## 4. Results

### 4.1. Verification of Force Measurement

In the following, the signals acquired by the presented smart tool holder during milling and drilling tests are compared to the force signals measured by the stationary dynamometer. First, the dynamometer signals of the milling tests are pre-processed for verification of the bending moment measurement. Since the dynamometer measures forces in stationary Cartesian coordinates (X, Y, Z), while the tool holder measures bending moments in rotating tool coordinates, direct comparison of these raw signals is not feasible. Instead, an equivalent bending moment signal ($M_{b,dyn}\left(t\right)$) needs to be calculated from the dynamometer data in order to have a reference signal whose relative changes can be utilized for comparison. Therefore, the absolute force values in the X-Y plane ($F_{xy}\left(t\right)$) are calculated by superposition of the measured force signals in the X and Y directions ($F_{x}\left(t\right)$ and $F_{y}\left(t\right)$, respectively) according to the following equation:

(9) $$F_{xy}\left(t\right)=\sqrt{{\left(F_{x}\left(t\right)\right)}^{2}+{\left(F_{y}\left(t\right)\right)}^{2}}.$$

By multiplication with the tool length ($l_{tool}=174\;mm$), the equivalent bending moment signal of the dynamometer ($M_{b,dyn}\left(t\right)$) is determined by

(10) $$M_{b,dyn}\left(t\right)=F_{xy}\left(t\right)*l_{tool}.$$

**Figure 7:**
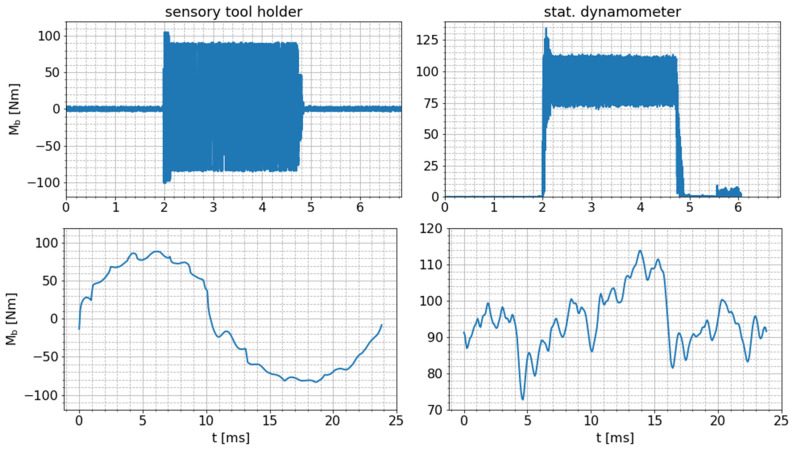
Comparison of raw bending moment signals measured by the sensory tool holder and bending moment extracted from raw dynamometer signals for one exemplary milling test with tooth feed of $f_{z}=0.25\;mm$ (top: whole process; bottom: one exemplary tool revolution).

In Figure 7, the raw bending moment signals measured by the smart tool holder ($M_{b}\left(t\right)$) and the bending moment ($M_{b,dyn}\left(t\right)$) extracted from the stationary dynamometer signals are presented exemplarily for one milling process and one tool revolution during this process.

Notably, the signal pattern differs between the two measurements. This is due to the fact that the measurement direction of the dynamometer is fixed in the stationary workpiece coordinate system, while the measurement direction of the smart tool holder signal is fixed in the rotating tool coordinate system. As a consequence, the bending moment measured via the smart tool holder varies nearly sinusoidally around zero between positive and negative maximum values. In contrast, due to the stationarity of the dynamometer with respect to the workpiece, the dynamometer signal reaches a certain non-zero bending moment level during the cutting process. The signal fluctuations around this level are caused by the consecutive engagement and disengagement of different cutting edges in the workpiece material, including different maxima of the bending moment per tooth due to tool runout (small differences in the local radii of the cutting teeth).

Further comparison is enabled by the maximum amplitudes of the bending moment signals. The amplitude (${\hat{M}}_{b}$) of the sensory tool holder signal is calculated for consecutive time windows (n) of length $∆t=t_{n-1}-t_{n}=0.05\;s$ according to

(11) $${\hat{M}}_{b}\left(n\right)=\frac{\max\left(M_{b}\left(t\right)\right)-\min\left(M_{b}\left(t\right)\right)}{2},\;t\in [t_{n-1},\;t_{n}].$$

The maximum values of the dynamometer signal ($M_{b,dyn,max}$) are calculated for the same consecutive time windows as in Equation (11) via

(12) $$M_{b,dyn,max}\left(n\right)=\max\left(M_{b,dyn}\left(t\right)\right),\;t\in [t_{n-1},\;t_{n}].$$

The maximum value of the dynamometer measurement corresponds to the maximum bending value that is applied to the tool. The amplitude (${\hat{M}}_{b}$) measured via the smart tool holder is typically smaller than the maximum bending moment ($M_{b,dyn,max}$) measured via the dynamometer. This is due to the fact that at the time when the maximum radial cutting force associated with the maximum bending moment ($M_{b,dyn,max}$) occurs, the bending moment measurement direction of the rotating smart tool holder is typically not equivalent to the direction of the maximum radial cutting force. The ratio (${\hat{M}}_{b}/M_{b,dyn,max}\leq 1$) between these two measured values depends on the concrete relative orientation between the cutting tool and the tool holder and the considered cutting conditions that specify the direction of the maximum radial forces acting on the tool.

**Figure 8:**
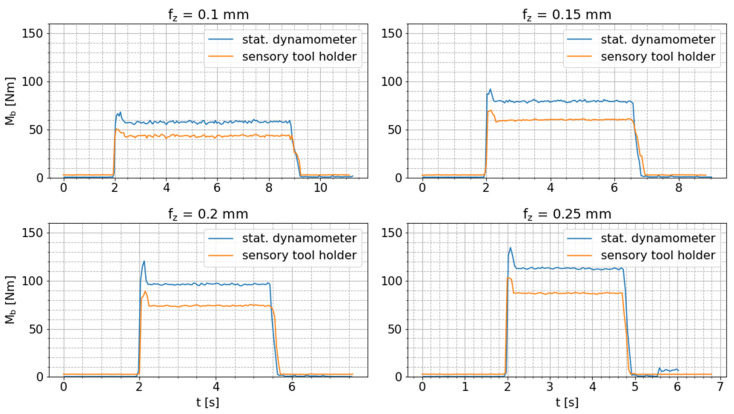
Comparison of amplitude of bending moment signal measured by the sensor-integrated tool holder and maximum bending moment measured by the stationary dynamometer during milling tests with varying feed per tooth ($f_{z}$).

As the orientation between the tool holder and the cutting tool is kept constant for all milling tests and the cutting kinematics are only slightly changed due to increasing feed rates, the ratio (${\hat{M}}_{b}/M_{b,dyn,max}$) between the amplitude of the tool holder signal and the maximum bending moment measured via the dynamometer should be approximately constant for all tests and not change over time if the tool holder-integrated bending moment sensor is correctly functioning. The two pre-processed bending moment signals are presented in Figure 8. These results show that the amplitudes of the tool holder signal stay within the range of the raw dynamometer signal, i.e., ${\hat{M}}_{b}\leq M_{b,dyn,max}$. Moreover, the amplitudes of the tool holder signal change over time in the same way as the maximum dynamometer signal.

The mean values of ${\hat{M}}_{b}$ and $M_{b,dyn,max}$ and the mean ratio (${{\hat{M}}_{b}/M}_{b,dyn,max\;}$) for the cutting processes are presented in Table 4. The ratio (${{\hat{M}}_{b}/M}_{b,dyn,max}$) is approximately constant for all tests. The small increase in this ratio might be caused by the slightly changing cutting kinematics due to the increasing feed rate and, thus, changing angular orientation of the maximum radial cutting force. Nevertheless, the described intended characteristics of the tool holder-integrated bending moment measurement are thereby confirmed, and its fundamental functionality is verified.

**Figure 9:**
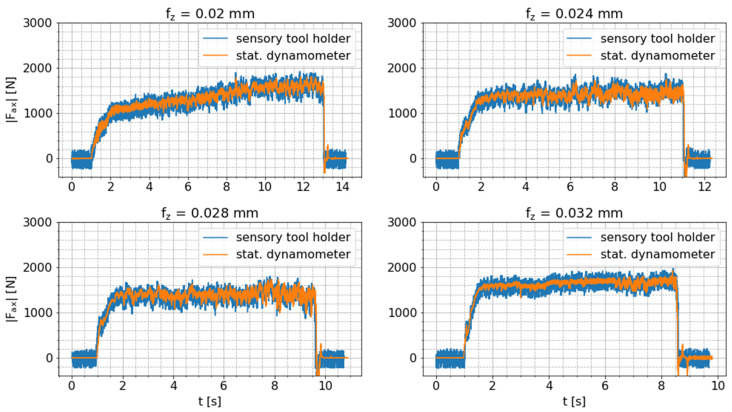
Comparison of raw axial force signals measured by sensory tool holder and stationary dynamometer during drilling tests with varying feed per tooth $f_{z}$.

For verification of the tool holder-integrated axial force measurement, drilling tests are conducted. In contrast to the bending moment measurement, conversion of the dynamometer signals is not required, since the force measured in the Z direction by the dynamometer does not change its orientation in relation to the axial force measured by the smart tool holder, despite the rotational movement. Just the sign of these force signals is reversed due to the reversed orientation of the corresponding coordinate systems. Hence, the absolute values of these signals are compared. Figure 9 shows the raw axial force signals of both sensor systems for all drilling tests with varying feed per tooth ($f_{z}$).

**Figure 10:**
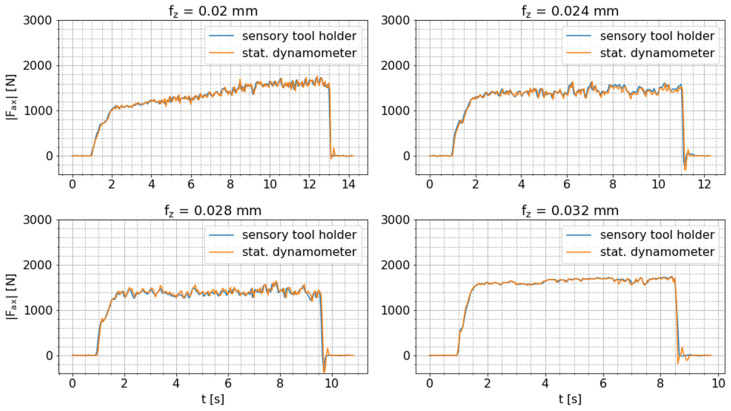
Comparison of mean axial force measured by the sensor-integrated tool holder and the stationary dynamometer during drilling tests with varying feed per tooth ($f_{z}$).

The raw measurement data of both sensor systems correlates well, although the tool-holder signal is superimposed by a high-frequency oscillation that is especially perceptible before and after the drilling process. In order to obtain a more reliable presentation of the correlation between these signals, the mean signal values (${\bar{F}}_{ax}$) of the raw axial force signals ($F_{ax}\left(t\right)$) are calculated in consecutive time windows (n) of length $∆t=t_{n-1}-t_{n}=0.05\;s$ according to following equation:

(13) $${\bar{F}}_{ax}\left(n\right)=\frac{1}{k}\sum_{t=t_{n-1}}^{t_{n}}F_{ax}\left(t\right),$$

where k is the number of signal values per window. In the case of the smart tool holder, the number of signal values (k) varies depending on the package loss of the BLE connection. Figure 10 shows these pre-processed mean axial force signals per sensor system and confirms the already determined good correlation between these signals. This comparison verifies the correct functionality of the tool holder-integrated axial force measurement.

### 4.2. Sensor Signal Characteristics

**Figure 11:**
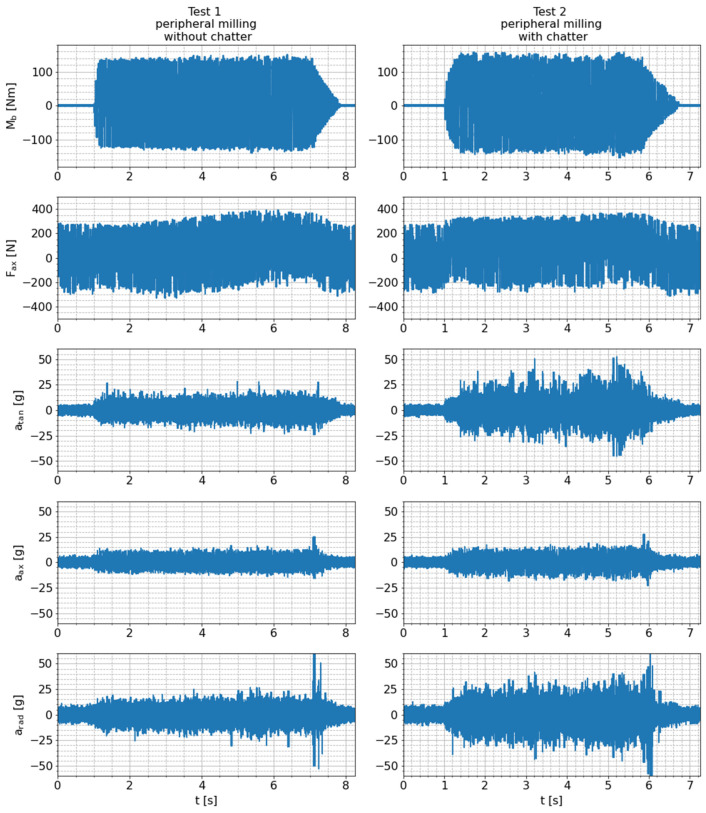
Raw sensor signals measured during tests 1 and 2.

**Figure 12:**
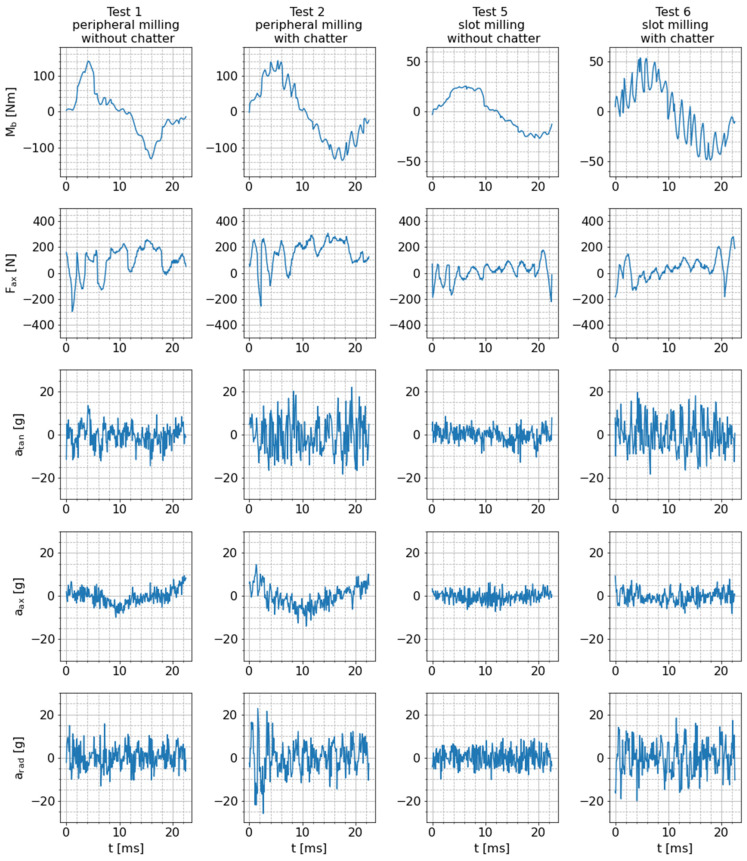
Raw sensor signals for one exemplary tool revolution during tests 1, 2, 5 and 6.

To obtain an overview of the fundamental sensitivity and suitability of the smart tool holder with its integrated sensors for process monitoring, at first, the raw sensor signals are analyzed. Therefore, all raw measurement data are exemplarily shown for test 1 (peripheral milling without chatter) and test 2 (peripheral milling with chatter) in Figure 11. As expected, the signal amplitudes are higher overall in test 2 than in test 1 because the depth of cut in test 2 is higher than in test 1 (12 mm in test 2 vs. 8 mm in test 1). Signal sequences of one exemplary tool revolution during tests 1 and 2 are compared in Figure 12 to obtain detailed insight into the signal characteristics during milling. Signal sequences of such exemplary tool revolutions are also shown for test 5 (slot milling without chatter) and test 6 (slot milling with chatter) in order to visualize the influence of a different milling strategy (lower cutting depth, maximum cutting width) on the raw sensor signals. Based on these figures, the fundamental behavior of the sensor signals during milling can be characterized as follows.

As already explained in Section 4.1, due to varying process forces and the fixed measurement direction of the bending moment on the rotating tool holder, the corresponding signal oscillates periodically around zero with the tool rotation frequency. An ideal sinusoidal oscillation would occur for constant radial cutting forces in the stationary workpiece coordinate system. However, due to small radial immersion of the milling tool resulting in a stronger variation of the radial cutting force component (tests 1 and 2) and superimposed high-frequency chatter vibrations (test 2 and 6), the measured signal shape deviates from the ideal sinusoidal form. In general, the periodic excitation of the tool is higher for tests with small radial immersion (tests 1 and 2), leading to higher amplitudes of the bending moment (140 Nm in tests 1 and 2 compared to 25 Nm in test 5 and 50 Nm in test 6). The different scaling of the y axis is also the reason why the superimposed chatter oscillations are more noticeable in test 6 than in test 2.

The axial force signals shown in Figure 12, on the other hand, are substantially superimposed by a seemingly periodic component with a specific pattern and an amplitude of roughly 200 N. This signal component has a similar frequency, amplitude and pattern during both machining and free rotation (see also Figure 9). Hence, it is not considered to contain relevant information about the process. Due to the nature of this interfering signal component, it is unlikely to be noise originating from signal processing hardware. Instead, an insufficient strain-gauge application is a potential reason for this characteristic, e.g., if the adhesive layer is too thick and the strain gauges, therefore, obtain an additional degree of freedom, process-unrelated oscillations of the sensors could be the consequence. However, larger quasi-static axial forces can be resolved very well, which can be seen in the offset of the sensor signals in tests 1 and 2, indicating the quasi-static pulling and pushing forces acting on the tool in the peripheral milling tests (see also Figure 10).

The tangential, axial and radial acceleration signals measured by the integrated piezoelectric sensor show a considerably different characteristic compared to the strain gauge-based signals. The raw acceleration signals in all three sensing directions do not comprise distinctive frequency components, as is the case with the bending moment signal. Instead, they are similar to white noise but with an increased amount and amplitude of aperiodic signal peaks during the cutting process. This occurs only during rotation of the system, which leads to the assumption that the high centrifugal accelerations that statically act on the off-center applied piezoelectric sensor change its sensing behavior. In addition, one can see that the overall amplitude of the vibration signal is slightly larger for milling with chatter (test 2) compared to dynamically stable cutting (test 1).

### 4.3. Capability of Sensors for Chatter Detection

To extract clear process-related information from the sensors of the smart tool holder, the raw sensor signals are pre-processed. Here, we focus on straightforward approaches for chatter detection. A general overview and more sophisticated and specialized methods for chatter detection can be found in the review article reported in [26].

From the bending moment signals in the time domain, two specific signal features are calculated. The first feature is the amplitude (${\hat{M}}_{b}$) of the bending moment signal ($M_{b}\left(t\right)$), which is calculated as described in Section 4.1. Due to the nature of data transmission via BLE and maximum utilization of the microcontroller, package loss appears occasionally, resulting in discontinuous sensor signals. Analysis of such signals in the frequency or time–frequency domain in their entirety is not feasible. For this reason, just the continuous signal sequences per package are pre-processed to extract the chatter-relevant high-frequency information. This is realized via discrete wavelet transformation (DWT), as described in [27], using a Daubechies-5 wavelet. In particular, the bending moment signals per data package (length of 32 values) are decomposed into two signal components, known as detail (wavelet) coefficients ($c_{D}$) and approximation (scaling) coefficients ($c_{A}$). While the approximation coefficients contain the low-frequency signal part, the detail coefficients contain the high-frequency signal part and, hence, are of interest for bending moment signal analysis. This is done on multiple levels, meaning that in the next level, the approximation coefficients from the previous level are, again, decomposed into new detail and approximation coefficients. As a consequence, detail coefficients of lower decomposition levels refer to higher-frequency components. The detail coefficients of the data package (p) at decomposition level i are denoted by $c_{D,p,i}$. To obtain an indicator of the signal energy in different frequency bands, the signal energy ($E_{c_{D},i}$) of the detail coefficients ($c_{D,p,i}$) is calculated via the following equation:

(14) $$E_{c_{D},i}\left(p\right)=\sum_{l=0}^{L_{i}}{\left|c_{D,p,i}\left(l\right)\right|}^{2},$$

where $L_{i}$ is the number of detail coefficients at the *i*th decomposition level. In order to establish a convenient second feature of the bending moment signal for chatter detection, the mean energy (${\bar{E}}_{c_{D},i}$) within consecutive time windows (n) of length $∆t=t_{n-1}-t_{n}=0.05\;s$ is calculated by

(15) $${\bar{E}}_{c_{D},i}\left(n\right)=\frac{1}{p_{n}-p_{n-1}}\sum_{p=p_{n-1}}^{p_{n}}E_{c_{D},i}\left(p\right),$$

where $p_{n}$ specifies the package number at time step $t_{n}$. Since the detail coefficients of two DWT decomposition levels (i=2,3) are significant regarding chatter, their mean energy values per window are summed as

(16) $${\bar{E}}_{c_{D},2+3}\left(n\right)={\bar{E}}_{c_{D},2}\left(n\right)+{\bar{E}}_{c_{D},3}\left(n\right).$$

In the previous section, it was shown that analysis of frequency components or amplitudes of the axial force signals is not feasible. Only the signal offsets during milling processes potentially contain significant information. To extract this offset, the mean values (${\bar{F}}_{ax}$) of the axial force signals are calculated in consecutive time windows, as described in Section 4.1.

As mentioned above, frequency components of the triaxial acceleration signals are also insignificant for process monitoring. Therefore, the signals are not transformed into the frequency or time-frequency domain. Instead, each signal ($a\left(t\right)$) is evaluated in the time domain by calculation of the root mean square ($a_{RMS}$) in the same consecutive time windows (n) as for the sensor signal features (${\hat{M}}_{b}$, ${\bar{E}}_{c_{D},2+3}$ and ${\bar{F}}_{ax}$) as follows:

(17) $$a_{RMS}\left(n\right)=\sqrt{\frac{1}{k}\sum_{t=t_{n-1}}^{t_{n}}{\left(a\left(t\right)\right)}^{2}}.$$

This feature extracts the relevant information provided by the varying amount and amplitude of the aperiodic signal peaks.

**Figure 13:**
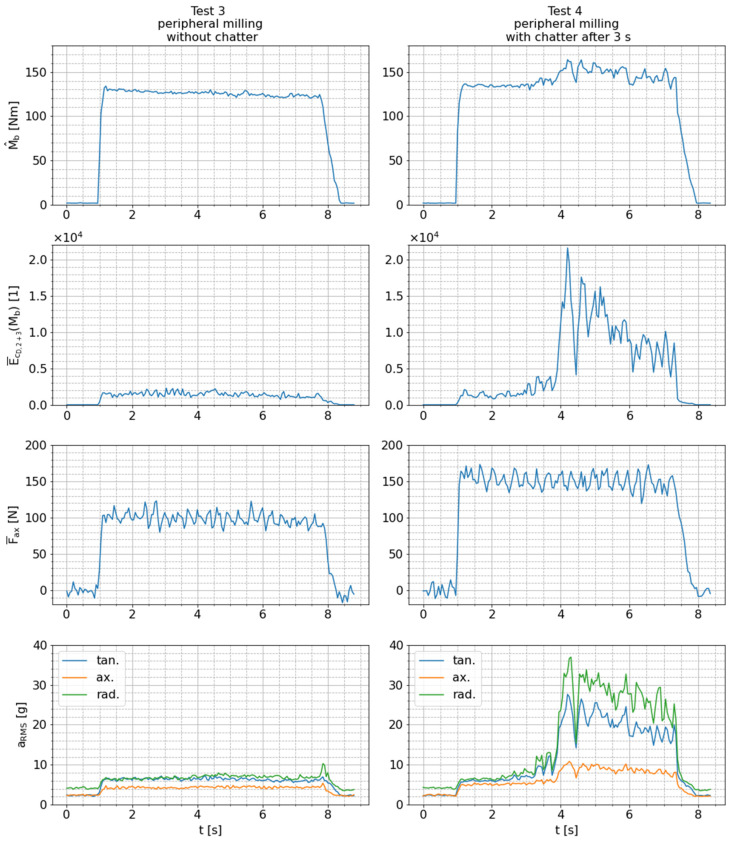
Features extracted from raw sensor signals measured during tests 3 and 4.

In order to estimate their general significance regarding chatter detection, the extracted features per sensor signal are presented for test 3 (peripheral milling without chatter) and test 4 (peripheral milling with chatter) in Figure 13. As described in Section 3, chatter only occurs in the second half of the cutting process during test 4. This circumstance allows for analysis of the following two phenomena:

- The separated influence of higher cutting depth on the signal features when comparing test 3 and the first half of test 4 and
- The isolated influence of chatter occurrence while milling with identical cutting parameters when comparing the first and second halves of test 4.

Because of the larger cutting depth, the signal feature values (${\hat{M}}_{b}$ and $a_{RMS}$) for the axial sensing direction are slightly increased in the first half of test 4 compared to test 3. The values of these features further increase in the second half of test 4 solely due to chatter. Dynamic instabilities can be identified even more clearly in the signal features (${\bar{E}}_{c_{D},2+3}$ and $a_{RMS}$) for the tangential and radial sensing directions. At the beginning of test 4, their values are at the same level as in test 3, despite the greater depth of cut. Therefore, these features appear to be independent of this cutting parameter. When chatter starts in the second half of test 4, however, the feature values increase substantially. This characteristic enables sensitive detection of chatter. The side-by-side comparison in Figure 13 also demonstrates that the increase in the mean axial force (${\bar{F}}_{ax}$) is only caused by the greater depth of cut, since there is no change in the case of chatter. For that reason, this signal feature is not suitable as a chatter indicator and is not further investigated. The bending moment amplitude (${\hat{M}}_{b}$) is also not further investigated because the relative increase in this signal feature due to chatter is marginal compared to the increase in the other presented features. Furthermore, ${\hat{M}}_{b}$ correlates with the radial forces applied to the tool and, therefore, heavily depends on the selected set of cutting parameters, which is not suitable for a chatter indicator.

**Figure 14:**
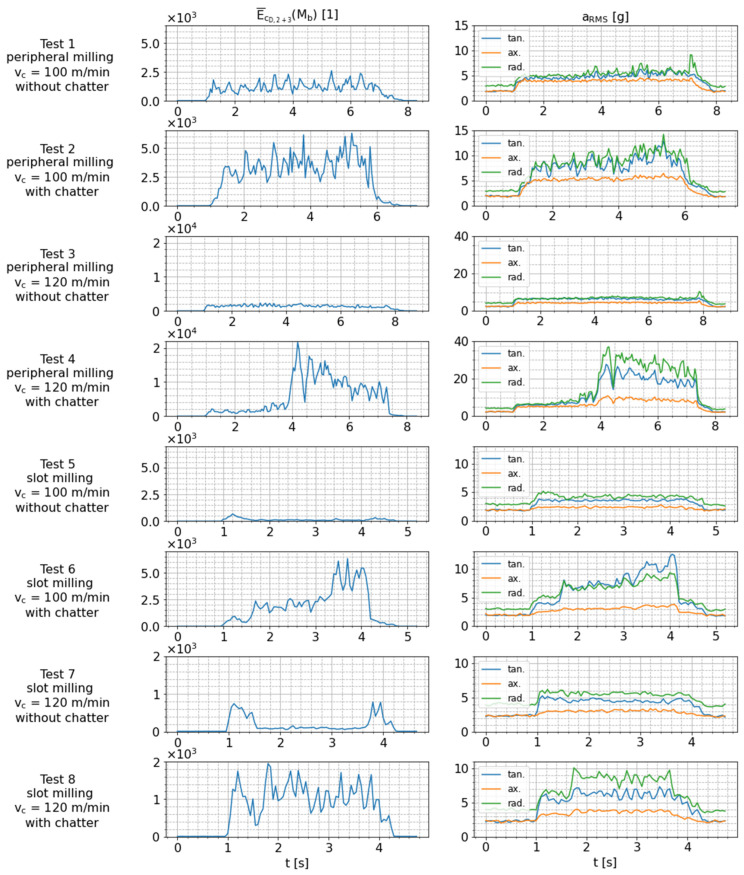
Comparison of chatter-sensitive features extracted from raw sensor signals measured during tests 1 to 8.

The remaining signal features (${\bar{E}}_{c_{D},2+3}$ and $a_{RMS}$) that are relevant to detect chatter are presented in Figure 14 for all conducted milling tests. Note that two tests (two consecutive rows) are always performed with the same milling strategy and cutting speed (cf. Table 3), which means that in these tests, the isolated influence of chatter on the features directly shows up (e.g., comparisons between tests 1 and 2, between test 3 and 4 etc.). The observations that were previously described for peripheral milling in tests 3 and 4 (Figure 13) also apply to the data for the other tests. The mean detail coefficient energy of the bending moment signal (${\bar{E}}_{c_{D},2+3}$), as well as the root mean square ($a_{RMS}$) of the radial and tangential acceleration signals, is significantly increased in case of chatter (tests 2, 4, 6 and 8). The $a_{RMS}$ values for the axial sensing direction, however, are only slightly increased. This means that the tangential and radial acceleration signals, as well as the bending moment signal, are highly sensitive to the occurrence of chatter. Therefore, the suitability of the smart tool holder for chatter detection is confirmed.

As described in Section 3, chatter is less intense in test 8 than in test 6 due to the difference in cutting speed (spindle speed) and consequent differences in dynamical behavior of the mechanical structure. However, the $a_{RMS}$ values for the radial and axial sensing directions are at similar levels in tests 6 and 8. In contrast, the values of ${\bar{E}}_{c_{D},2+3}$ and $a_{RMS}$ for the tangential sensing direction are notably higher in test 6 than in test 8, which is in accordance with the perceived higher level of chatter intensity and might indicate better sensitivity regarding this effect.

Furthermore, when comparing the dynamically stable slot milling tests 5 and 7 to the dynamically stable peripheral milling tests 1 and 3, a similar level of $a_{RMS}$ for all sensing directions is identified. This indicates only a small dependence of the acceleration signals on the cutting parameters and milling strategies, which is favorable for the usage of these signals as a general chatter indicator. On the other hand, the values of ${\bar{E}}_{c_{D},2+3}$ for the slot milling tests tests 5 and 7 are significantly lower than for the peripheral milling tests (tests 1 and 3). This is due to the fact that the excitation of the milling tool with changing radial forces is much higher for peripheral milling with low radial immersion than for slot milling with nearly constant cutting forces in the case of stable cutting.

## 5. Conclusions

In this work, a multi-sensory tool holder for sensitive monitoring of milling processes is presented. Sensor integration involves strain gauges for axial and radial force measurement, a piezoelectric accelerometer for monitoring of triaxial vibrations and a resistive temperature sensor. This paper describes the application and functionality of the sensors, as well as the design of the sensor-integrated tool holder without interfering contours. The sensors are connected to a printed circuit board (PCB) for signal processing, which includes amplification, filtering and digitalization. A novel electric generator is utilized for the tool holder’s energy supply by converting kinetic energy from tool rotation into electrical energy through electromagnetic induction. Therefore, permanent operation of the system completely independent from external energy sources is enabled. Transmission of digitalized sensor data to a PC unit is realized wirelessly using the Bluetooth Low Energy (BLE) protocol.

Experimental milling and drilling tests are conducted on two machine tools with various cutting parameters to generate a meaningful set of measurement data. The functionality of the strain gauge-based force measurement of the smart tool holder is verified by comparison with the forces measured by a stationary dynamometer. Furthermore, the sensors’ fundamental behavior during milling and their ability to detect dynamic instabilities (chatter) are investigated.

The results show that the bending moment signal provides the most information about the milling process. It contains information about the varying radial forces applied to the tool due tool rotation, individual cutting-edge engagements and chatter. A reliable chatter detection feature is derived from this signal via discrete wavelet transformation (DWT) by analyzing the mean signal energy corresponding to high-frequency detail coefficients. The axial force signal clearly indicates the quasi-static pulling and pushing forces applied to the tool during milling and drilling processes but is not suitable for a detailed analysis of the cutting process due to interfering noisy oscillations. Similarly, noisy oscillations caused by sensor rotation disturb the triaxial acceleration signals. Therefore, a detailed frequency or time–frequency analysis of accelerations during the cutting process is not feasible. However, the root mean square (RMS) of the radial and, especially, the tangential acceleration is a clear indicator of the occurrence of chatter vibrations in end milling.

In future work, further experiments with different types and sizes of cutting tools, as well as different workpiece materials and cutting parameters, will be conducted, and the suitability of the tool holder-integrated sensors for other monitoring objectives, such as monitoring of tool wear and the detection of tool breakage, will be investigated. Moreover, mechanical properties of the tool holder system, such as static and dynamic stiffness or natural frequencies, will be determined and compared to other sensor-integrated tool holders.

## Figures and Tables

**Table 1:** Parameters of bridge conversion equations.

Parameter	Bending Moment Bridge	Axial Force Bridge
Gauge factor (k)	2.18	2.26
Poisson’s ratio (ν)	–	0.126
Proportionality factors (Π_b, Π_ax)	5.36 × 10^−7^ (Nm)^−1^	1.01 × 10^−8^ N^−1^
$\mathrm{Bridge}\;\mathrm{supply}\;\mathrm{voltage}\;(U_{B}$)	9 V	9 V

**Table 2:** Sensor and signal processing properties.

Sensor Signal	Measurement Range	Resolution	Sampling Rate	Low-Pass Filtration Frequency
$\mathrm{Bending}\;\mathrm{moment}\;(M_{b}$)	±400 Nm	0.2 Nm	10 kHz	2.4 kHz
$\mathrm{Axial}\;\mathrm{force}\;(F_{ax}$)	±15 kN	7.5 N	10 kHz	2.4 kHz
$\mathrm{Acceleration}\;\mathrm{in}\;\mathrm{radial},\;\mathrm{axial}\;\mathrm{and}\;\mathrm{tangential}\;\mathrm{directions}\;(a_{rad},\;a_{ax},\;a_{tan}$)	±100 g	0.08 g	10 kHz	5 kHz
Temperature (T)	−55–135 °C	0.02 °C	312.5 Hz	−

**Table 3:** Cutting parameters of milling tests for process monitoring.

Test No.	$\mathrm{Cutting}\;\mathrm{Speed}\;(v_{c}$) [m/min]	$\mathrm{Tooth}\;\mathrm{Feed}\;(f_{z}$) [mm]	$\mathrm{Width}\;\mathrm{of}\;\mathrm{Cut}\;(a_{e}$) [mm]	$\mathrm{Depth}\;\mathrm{of}\;\mathrm{Cut}\;(a_{p}$) [mm]	Chatter
1	100	0.07	3	8	no
2	100	0.07	3	12	yes
3	120	0.07	3	8	no
4	120	0.07	3	12	yes
5	100	0.05	12	1.0	no
6	100	0.05	12	1.5	yes
7	120	0.05	12	1.0	no
8	120	0.05	12	1.5	yes

**Table 4:** Mean pre-processed bending moment signals of sensory the tool holder and dynamometer and their relative difference per test with varying tooth feed.

$\mathrm{Tooth}\;\mathrm{Feed}\;(f_{z}$) [mm]	$\mathrm{Mean}\;\mathrm{Value}\;\mathrm{of}\;M_{b,dyn,max}$ [Nm]	$\mathrm{Mean}\;\mathrm{Value}\;\mathrm{of}\;{\hat{M}}_{b}$ [Nm]	$\mathrm{Ratio}\;({\hat{M}}_{b}/M_{b,dyn,max}$) [%]
0.10	57.53	43.24	75.2
0.15	79.33	60.04	75.7
0.20	96.04	73.80	76.8
0.25	112.56	86.81	77.1

## Data Availability

Data are contained within the article; further inquiries can be directed to the corresponding author.
